# Distinctive interactions of the *Arabidopsis *homolog of the 30 kD subunit of the cleavage and polyadenylation specificity factor (AtCPSF30) with other polyadenylation factor subunits

**DOI:** 10.1186/1471-2121-10-51

**Published:** 2009-07-02

**Authors:** Suryadevara Rao, Randy D Dinkins, Arthur G Hunt

**Affiliations:** 1 Department of Plant and Soil Sciences, University of Kentucky, Lexington, KY 40546-0312 USA; 2 USDA-ARS, FAPRU, Lexington, KY 40546-0091 USA

## Abstract

**Background:**

The *Arabidopsis *ortholog of the 30 kD subunit of the mammalian Cleavage and Polyadenylation Specificity Factor (AtCPSF30) is an RNA-binding endonuclease that is associated with other *Arabidopsis *CPSF subunits (orthologs of the 160, 100, and 73 kD subunits of CPSF). In order to further explore the functions of AtCPSF30, the subcellular distribution of the protein was examined by over-expressing fusion proteins containing fluorescent reporters linked to different CPSF subunits.

**Results:**

It was found that AtCPSF30 by itself localizes, not to the nucleus, but to the cytoplasm. AtCPSF30 could be found in the nucleus when co-expressed with AtCPSF160 or AtCPSF73(I), one of the two *Arabidopsis *orthologs of CPSF73. This re-directing of AtCPSF30 indicates that AtCPSF30 is retained in the nucleus via interactions with either or both of these other CPSF subunits. Co-expression of AtCSPF30 with AtCPSF100 altered the location, not of AtCPSF30, but rather of AtCPSF100, with these proteins residing in the cytoplasm. Deletion of plant-specific N- or C-terminal domains of AtCPSF30 abolished various of the interactions between AtCPSF30 and other CPSF subunits, suggesting that the plant CPSF complex assembles via novel protein-protein interactions.

**Conclusion:**

These results suggest that the nuclear CPSF complex in plants is a dynamic one, and that the interactions between AtCPSF30 and other CPSF subunits are different from those existing in other eukaryotes.

## Background

Messenger RNA polyadenylation is mediated by a multifactor complex in eukaryotes. In mammals, this complex consists of the Cleavage Stimulatory Factor (CstF), the Cleavage and Polyadenylation Specificity Factor (CPSF), two Cleavage Factors (CFIm and CFIIm) and poly(A) polymerase [1-3]. CPSF binds to the AAUAAA sequence via its 160 kD subunit [4], and CstF recognizes the downstream element via its 64 kD subunit [5-7]. Poly(A) tail formation is further controlled by a nuclear poly (A)-binding protein [8]. The polyadenylation of pre-mRNAs occurs in the nucleus, and is coupled to the transcription process at many different steps.

Among the more interesting of the subunits of the polyadenylation complex is the 30 kD subunit of CPSF (CPSF30). CPSF30 proteins possess tandem arrays of 3–5 CCCH zinc finger motifs [9-14] that are involved in a number of functions of the proteins. CPSF30 from *Drosophila melanogaster *[11], mammals [10], yeast [9,10,15], and *Arabidopsis *[13,14] bind RNA. Moreover, the *Arabidopsis *and *Drosophila *CPSF30 proteins possess endoribonucleolytic activity [11-13]. The *Arabidopsis *CPSF30 (AtCPSF30) has been implicated in the responses of plants to oxidative stress [16], and is subject to controls *in vitro *that further suggest regulation by cellular redox status and heavy metal exposure [17].

AtCPSF30 lies at the center of a network of protein-protein interactions involving other polyadenylation factor subunits [18]. Among these interactions are those with two other CPSF subunits – AtCPSF160 and AtCPSF100. Beyond the documentation using the two-hybrid assay [18], the significance of these interactions has not been studied. In this report we extend these previous observations with studies of the subcellular location of tagged forms of AtCPSF30 in plant cells, using a transient expression assay that utilizes over-expression of tagged forms of putative interacting proteins. Our results reveal that, in the absence of other CPSF subunits, over-expressed AtCPSF30 is situated in the cytoplasm. In cells that co-express AtCPSF30 with either AtCPSF160 or AtCPSF73(I), AtCPSF30 can be found in the nucleus, suggesting that association with other CPSF subunits is responsible for the nuclear localization of AtCPSF30. Co-expression of AtCPSF30 with AtCPSF100 results in a re-localization of the latter two proteins to the cytoplasm. As AtCPSF100 by itself is nucleus-localized, this observation indicates that the interaction between AtCPSF30 and AtCPSF100 alters the distribution of AtCPSF100. Finally, we show that the interactions of AtCPSF30 with other CPSF subunits involve plant-specific domains of AtCPSF30. This suggests that the organization of the plant CPSF complex is different from the analogous complexes in other eukaryotes. These results permit a substantial revision of models of the CPSF complex in plants.

## Results

### AtCPSF30 does not appear to possess an inherent nuclear localization signal

To study the subcellular distribution of the *Arabidopsis *CPSF30, the AtCPSF30 coding region was fused to the C-termini of the DsRed2 or GFP genes (a list of the constructs used is provided in Table 1). Control constructs encoded unmodified DSRed2 [19], as well as fusion proteins containing the product of the *Arabidopsis *NDA2 gene (a mitochondrial marker; [20]), a synthetic endoplasmic reticulum localization sequence [21], and a zinc finger protein (AtZFP11) known to localize to the nucleus [22]. In all of these constructs, expression was under control of the cauliflower mosaic virus 35S promoter; the choice of overexpression using the 35S promoter was based on prior observations indicating that the levels of AtCPSF30 in wild-type plants are exceedingly low [16], and thus detection of the products of transgenes driven by the promoter from the AtCPSF30 gene (At1g30460) was not feasible. Overexpression in transient assays has been used by others to study protein localization and protein-protein interactions, and often reveals unexpected aspects of the functioning of protein complexes [23,24]. It was thus expected that this approach would provide information about the inherent subcellular location signals carried by AtCPSF30. Accordingly, various combinations of these plasmids were introduced into tobacco leaf cells using a biolistics apparatus and the protein expression and localization assessed using confocal microscopy.

**Table 1:** List of localization constructs used in this study

**Protein of interest**	**reporter**	**vector backbone**	**Source or citation**
AtCPSF30	DSRed2	pGD [19]	This study

AtCPSF30-m4	DSRed2	pGD [19]	This study

AtCPSF30-m9	DSRed2	pGD [19]	This study

AtCPSF30	GFP	pGD [19]	This study

AtCPSF160	GFP	pMBC43 [46]	Q. Q. Li unpublished

AtCPSF100	GFP	pMBC43 [46]	Q. Q. Li unpublished

AtCPSF73(I)	GFP	pKYLX80 [22]	[30]

AtCPSF73(II)	GFP	pKYLX80 [22]	[30]

AtZFP11	GFP	pKYLX80 [22]	[22]

AtZFP11	DSRed2	pKYLX80 [22]	[22]

NDA2	GFP	pKYLX80 [22]	[20]

ER*	GFP	pSITE [47]	[47]

AtDcp2	DSRed2	pGD [19]	This study

DSRed2	DSRed2	pGD [19]	[19]

**Figure 1:**
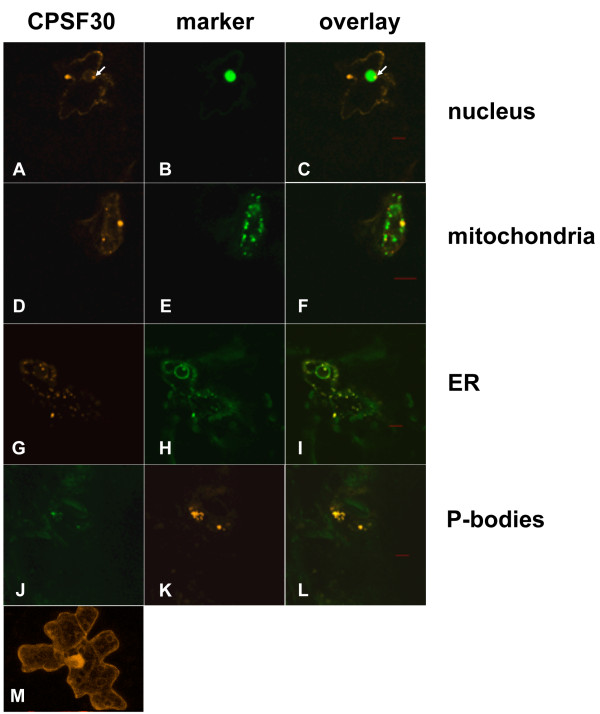
**Subcellular distribution of AtCPSF30 in tobacco cells**. In this figure, the overlays of the two images corresponding to pairs of fusion proteins are shown in the column on the right (panels C, F, I, and L). The AtCPSF30 is visualized in panels A, D, G, and J. Markers used to assess the subcellular location of AtCPSF30 were: nuclear marker (GFP-AtZFP11), panel B; mitochondrial marker (GFP-NDA2), panel E; endoplasmic reticulum marker (the synthetic localization sequences carried in the mgfp4-ER plasmid described by Haseloff *et al*. [21]), panel H; and the P-body marker (Dcp2), panel K. The distribution of unmodified DSR is shown in panel M. Red bars are 10 μm size markers.

In cells co-transfected with DSRed2-AtCPSF30 and GFP-AtZFP11, no accumulation of DSRed2-AtCPSF30 could be seen in the nucleus, (Figure 1, panels A-C). Instead, a distribution of DSRed2-AtCPSF30 outside of the nucleus, in distinct foci, was apparent. A similar pattern was not seen in cells that express unmodified DSRed2 (Figure 1, panel M). These foci did not correspond to chloroplasts (visualized using the fluorescent properties of the plastid; not shown). Moreover, they did not co-localize with the GFP-NDA2 marker (Figure 1, panels D-F), indicating that they were not mitochondrially-localized. These extranuclear foci were noticeably mobile (see Additional File 1), and were coincident with the endoplasmic reticulum (Figure 1, panels G-I). However, some locales of the ER marker (the nuclear envelope and nucleolus) were devoid of DSRed2-AtCPSF30. Invariably, while DSRed2-AtCPSF30 could not be seen in the nucleus to any appreciable extent, one or more of the cytoplasmic DSRed2-AtCPSF30-containing foci abutted the nucleus (as indicated with the arrows in panels A and C of Figure 1).

As an important cytoplasmic location for RNA metabolism is the so-called processing body, or P-body [25,26], the co-localization of AtCPSF30 with an *Arabidopsis *P-body marker was studied. For this, DSR was fused to the *Arabidopsis *Dcp2 protein-coding region and GFP was fused to AtCPSF30. Dcp2 is a component of the *Arabidopsis *P-body [27-29] and serves in this study as a marker for this structure. In cells co-expressing the DSRed2-AtDcp2 and GFP-AtCPSF30 fusion proteins, the DSRed2-AtDcp2 was found in distinctive foci (Figure 1, panel K), much as has been observed by others [28,29]. These foci were distinct from mitochondria and chloroplasts (not shown) in these cells. The DSRed2-AtDcp2 foci were coincident with the GFP-AtCPSF30 foci (Figure 1, panels J-L). This indicates that, in cells overexpressing the two proteins, GFP-AtCPSF30 and DSRed2-AtDcp2 are present in the same structures.

### The interaction between AtCPSF30 and AtCPSF160 promotes nuclear localization of AtCPSF30

Because messenger RNA 3' end formation is a nucleus-localized RNA processing event, the absence of DSRed2-AtCPSF30 from the nucleus was surprising. One possible explanation for this observation is that AtCPSF30 by itself possesses no nuclear localization information, but rather is recruited to the nucleus via interactions with other CPSF subunits. In this case, in cells transiently expressing DSRed2-AtCPSF30 from the 35S promoter, the protein might be present in a vast excess over other interacting partners and would thus localize to a "default" location in the cell. To explore this possibility, the DSRed2-AtCPSF30 fusion protein was co-expressed with GFP fusion proteins containing AtCPSF100, AtCPSF160, AtCPSF73(I), and AtCPSF73(II), respectively. AtCPSF160 and AtCPSF100 were chosen because they have been reported to interact with AtCPSF30 [18,30]. AtCPSF73(I) and AtCPSF73(II) are two relatives of the 73 kD subunit of CPSF in mammals and the yeast homolog Ysh1. While AtCPSF73(I) and AtCPSF73(II) apparently do not interact with AtCPSF30 in two-hybrid assays [18], they are part of the *Arabidopsis *CPSF complex and localize to the nucleus [30].

**Figure 2:**
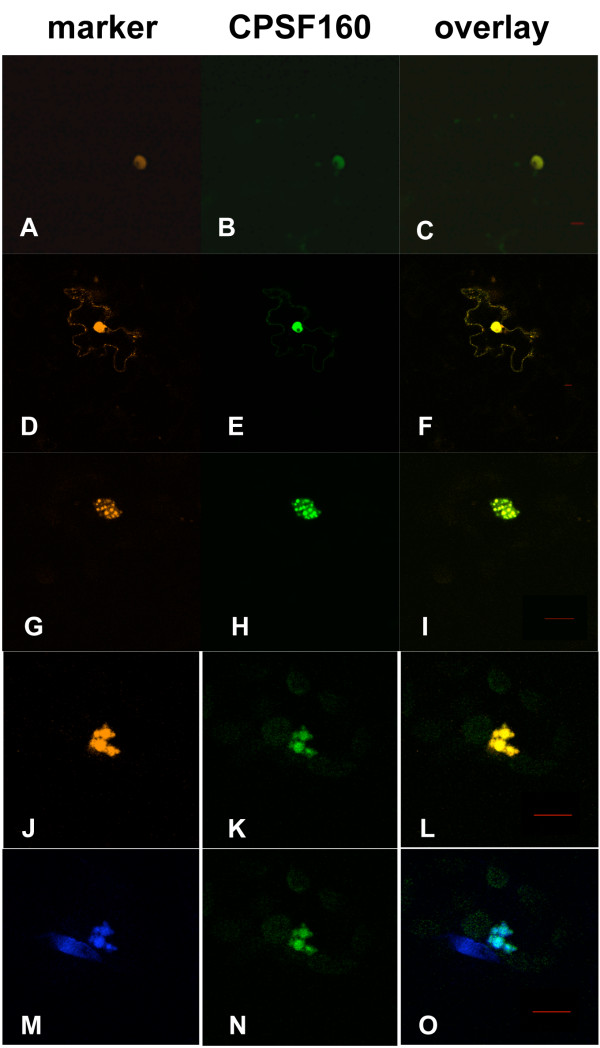
**Localization of AtCPSF30 in cells that also express AtCPSF160**. GFP-AtCPSF160 was co-expressed with DSRed2-AtZFP11 (panels A-C) or DSRed2-AtCPSF30 (panels D-O). The column labeled "CPSF160" denotes the images of the GFP-AtCPSF160 fusion protein, and the column labeled "marker" denotes either the DSRed2-AtZFP11 marker (panel A), DSRed2-AtCPSF30 (panels D, G, and J), or Hoechst stain (panel M). Panels K and N are the same sample; panel M shows the results of Hoechst staining of the cell shown in Panels J-O, and panel O is the overlay of panels M and N. Multiple cells expressing both AtCPSF30 and AtCPSF160 are shown, as are different scales, to illustrate the variations that were observed in these studies. Red bars in the overlay panels are 10 μm size markers.

In plant cells that co-express the GFP-AtCPSF160 and DSRed2-AtZFP11 fusion proteins, both proteins accumulated in the nucleus (Figure 2, panels A-C). This confirms the expected location of AtCPSF160 in plant cells, and indicates that AtCPSF160 possesses nuclear localization signals. Invariably, when GFP-AtCPSF160 was co-expressed with DSRed2-AtCPSF30, the latter accumulated exclusively in the nucleus (as did GFP-AtCPSF160; Figure 2, panels D-O). However, the distribution of these proteins within the nucleus was often different from that seen for GFP-AtCPSF160 by itself. In some cases (Figure 2, panels D-F), the distribution of the two proteins in the nucleus was similar to the nuclear marker (Figure 2, panel A). However, the more frequent result was that GFP-AtCPSF160 and DSRed2-AtCPSF30 co-localized to distinctive structures within the nucleus (two representative cells are shown in Figure 2, panels G-L); these structures also contained DNA, as they could be stained with Hoechst stain (Figure 2, panels M-O).

**Figure 3:**
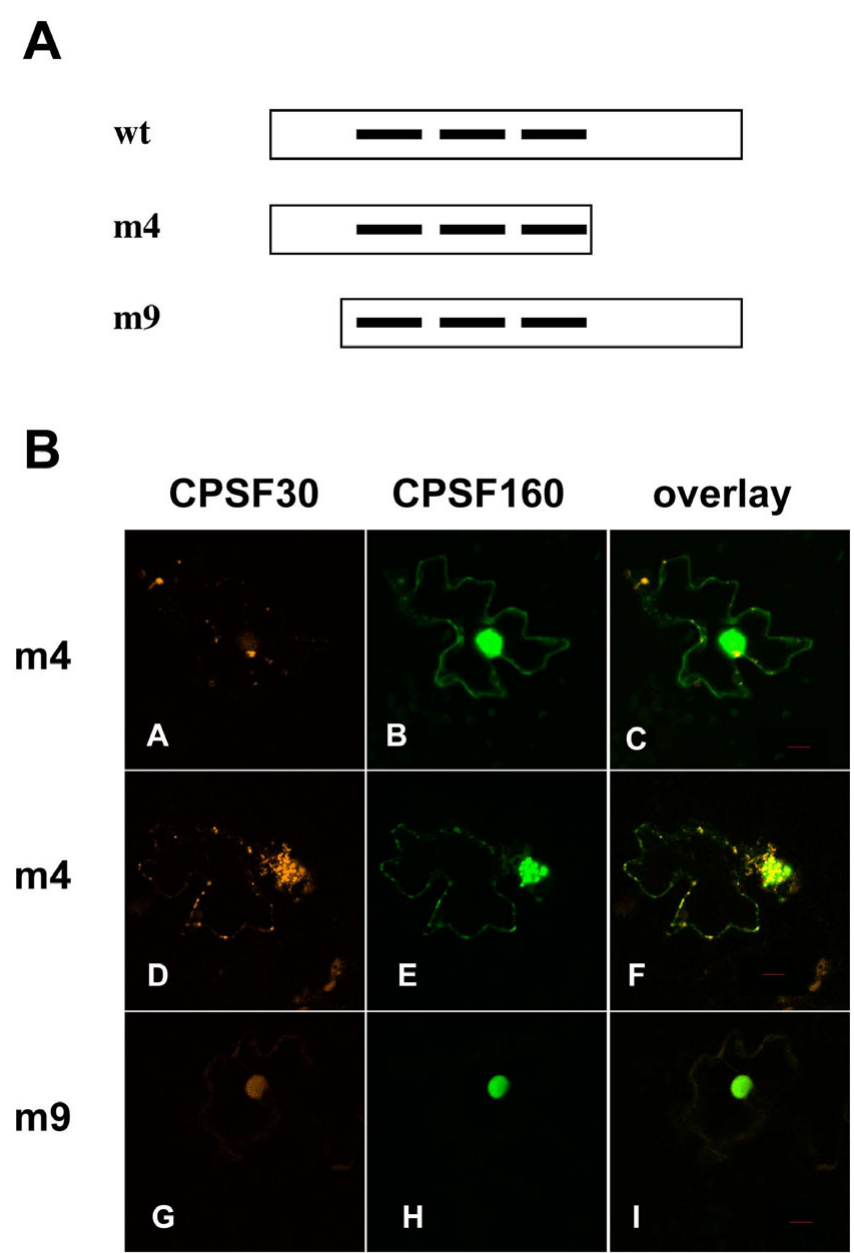
**Localization of mutant AtCPSF30 isoforms in the presence of AtCPSF160**. A. Illustration of the two deletion mutants (m4 and m9) used in this study; these mutants have been described in detail elsewhere [13,14]. B. Localization of fusion proteins in cells co-expressing either DSRed2-AtCPSF30 mutant as well as GFP-AtCPSF160. Two cells showing co-expressed DSRed2-m4 and GFP-AtCPSF160 are shown (panels A-F); these cells show the two variations into which all other examples may be grouped. A representative cell co-expressing the DSRed2-m9 mutant and GFP-AtCPSF160 is shown in panels G-I. Columns denote the protein being visualized (AtCPSF30, AtCPSF160, or the overlay). Red bars in the overlay panels are 10 μm size markers.

AtCPSF30 consists of three distinct domains [14] – a central core that includes the evolutionarily-conserved triad of CCCH zinc finger motifs that is flanked by novel N- and C-terminal domains (this general structure is illustrated in Figure 3A). To determine the part(s) of AtCPSF30 that are important for the interaction inferred by the effects of AtCPSF160 on AtCPSF30 localization, two deletion derivatives of AtCPSF30 were studied. These derivatives, termed m4 and m9 after Delaney *et al*. [14], lack either the C-terminal or N-terminal domains, respectively; together with the full-sized protein they permit an assignment of interactions to one of the three domains of the protein.

In cells expressing both GFP-AtCPSF160 and the DSRed2-m4 mutant, a range of results was obtained. In some cells (Figure 3B, panels A-C), GFP-AtCPSF160 was retained in the nucleus, while the DSRed2-m4 mutant protein displayed the subcellular distribution seen with DSRed2-AtCPSF30 in the absence of GFP-AtCPSF160 (as seen in Figure 1). In such cells, the distribution of GFP-AtCPSF160 in these nuclei was similar to that shown in Figure 2, panel B, as opposed to the concentration in subnuclear domains seen in Figure 2, panels H, K, and N. In some cells (Figure 3, panels D-F show a representative one), in addition to cytoplasmic DSRed2-m4, nuclear accumulation reminiscent of that seen in experiments performed with full-sized DSRed2-AtCPSF30 (Figure 2, panels G-L) was also seen. These results reveal a total (panels A-C) or partial (panels D-F) loss of the interaction between AtCPSF30 and AtCPSF160, such that some cytoplasmic DSRed2-m4 protein could be seen in cells co-expressing GFP-AtCPSF160. Thus, the C-terminal domain of AtCPSF30 seems to be important for efficient interactions with AtCPSF160.

In contrast, GFP-AtCPSF160 co-localized with the DSRed2-m9 mutant within the nucleus, and no extranuclear DSRed2-m9 mutant could be seen in cells co-expressing these two proteins (Figure 3, panels G-I). However, the novel subnuclear domains in which the two wild-type proteins accumulated were never seen in the experiments with the DSRed2-m9 mutant. Thus, the DSRed2-m9 mutant protein retains the ability to interact with GFP-AtCPSF160, but has lost the "ability" to change the subnuclear distribution of this protein. In the absence of AtCPSF160, the DSRed2-m4 and DSRed2-m9 variants were distributed in cells much as were the AtCPSF30 fusion proteins (not shown).

### Altered subcellular distributions in cells co-expressing AtCPSF30 with AtCPSF100 or AtCPSF73(I)

**Figure 4:**
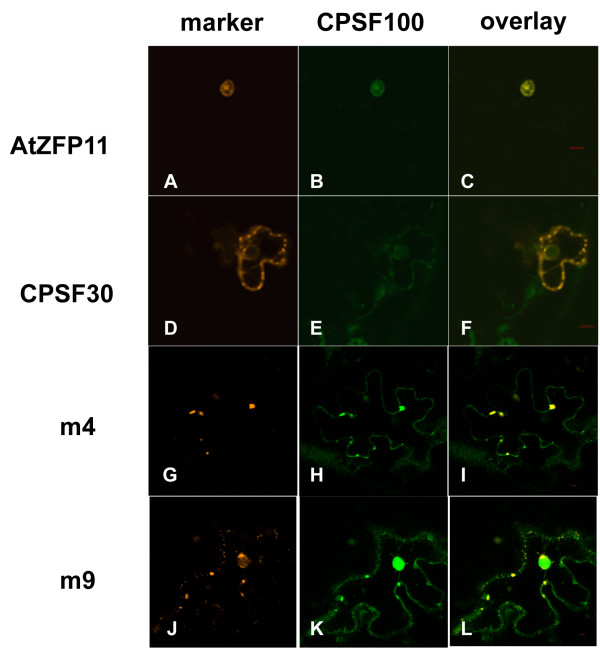
**Localization of AtCPSF30 and the m4 and m9 mutants in cells that also express AtCPSF100**. GFP-AtCPSF100 was co-expressed with DSRed2-AtZFP11 (panels A-C) or DSRed2 fused to AtCPSF30 (panels D-F), the m4 mutant (panels G-I), or the m9 mutant (panels J-L). The column labeled "CPSF100" denotes the images of the GFP-AtCPSF100 fusion protein, and the column labeled "marker" denotes either the DSRed2-AtZFP11 marker (panel A), DSRed2-AtCPSF30 (panel D), DSRed2-m4 (panel G), or DSRed2-m9 (panel J). Red bars in the overlay panels are 10 μm size markers.

Consistent with what has been reported elsewhere [30], GFP-AtCPSF100 co-localized with the nuclear marker (DSRed2-AtZFP11) when both were expressed in tobacco cells (Figure 4, panels A-C). Remarkably, co-expression of GFP-AtCPSF100 with DSRed2-AtCPSF30 changed the location of GFP-AtCPSF100, such that was it was largely in cytoplasmic foci (Figure 4, panel E). These foci also contained DSRed2-AtCPSF30 (Figure 4, panels D-F). This dramatic change in subcellular distribution of GFP-AtCPSF100 indicates that the interaction between this protein and AtCPSF30 [18] has the potential to interfere with the nuclear localization of AtCPSF100.

Similar results were obtained in cells co-expressing GFP-AtCPSF100 and the DSRed2-m4 variant (Figure 4, panels G-I). However, the nuclear localization of GFP-AtCPSF100 was largely restored in cells co-expressing the DSRed2-m9 variant along with GFP-AtCPSF100 (Figure 4, panels J-L). In these cases, the DSRed2-m4 and DSRed2-m9 variants remained in cytoplasmic foci, much as was seen with the wild-type DSRed2-AtCPSF30 (e.g., Figure 1) and with these two proteins when expressed without any CPSF partner (not shown). Thus, the redistribution of GFP-AtCPSF100 due to co-expression with DSRed2-AtCPSF30 requires the N-terminal part of AtCPSF30.

**Figure 5:**
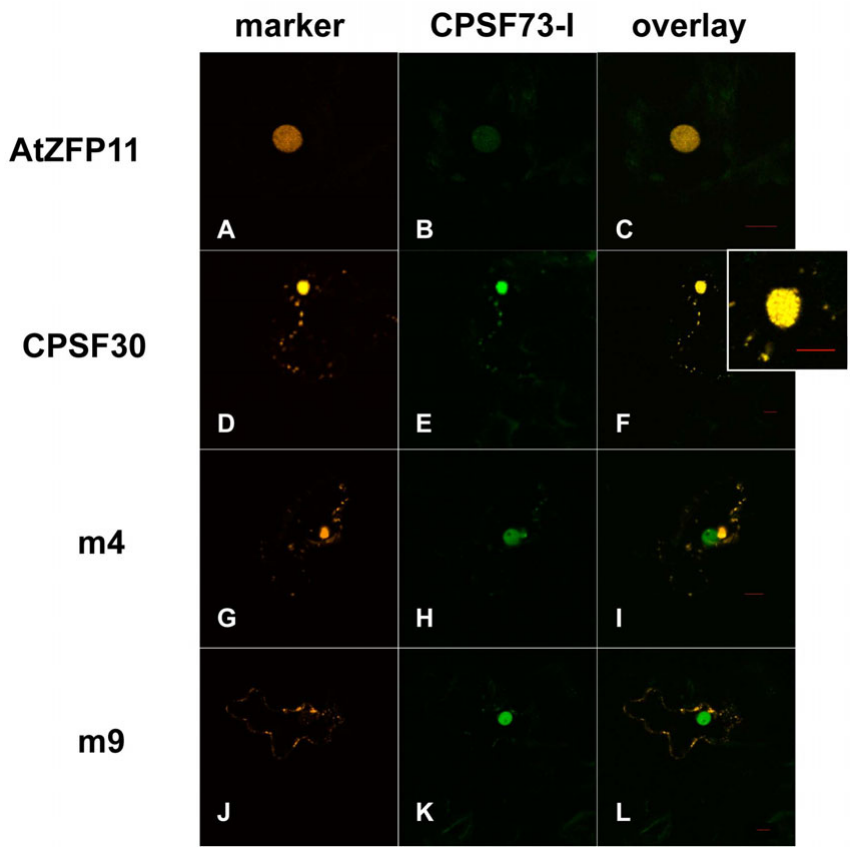
**Localization of AtCPSF30 and the m4 and m9 mutants in cells that also express AtCPSF73(I)**. GFP-AtCPSF73(I) was co-expressed with DSRed2-AtZFP11 (panels A-C) DSRed2 fused to AtCPSF30 (panels D-F), the m4 mutant (panels G-I), or the m9 mutant (panels J-L). The column labeled "CPSF73-I" denotes the images of the GFP-AtCPSF73(I) fusion protein, and the column labeled "marker" denotes either the DSRed2-AtZFP11 marker (panel A), DSRed2-AtCPSF30 (panel D), DSRed2-m4 (panel G), or DSRed2-m9 (panel J). The inset next to panel F shows a higher magnification of the cell in panel F, emphasizing the appearance of the nucleus. Red bars in the overlay panels are 10 μm size markers.

As was seen with GFP-AtCPSF160 and GFP-AtCPSF100, GFP-AtCPSF73(I) was located in the nucleus when co-expressed with the nuclear marker (Figure 5, panels A-C). In cells co-expressing GFP-AtCPSF73(I) and DSRed2-AtCPSF30, GFP-AtCPSF73(I) remained largely in the nucleus, although cytoplasmic foci containing GFP-AtCPSF73(I) were also discernible (Fig 5, panel E). In these cells, DSRed2-AtCPSF30 co-localized with GFP-AtCPSF73(I) in the nucleus and cytoplasm (Figure 5, panels D-F). Interestingly, nucleus-localized GFP-AtCPSF73(I) and DSRed2-AtCPSF30 accumulated in numerous large foci (inset of Figure 5, panel F).

In cells co-expressing the DSRed2-m4 variant along with GFP-AtCPSF73(I), the DSRed2-m4 protein was found in the cytoplasm but not the nucleus (Figure 5, panels G-I). In these cells, GFP-AtCPSF73(I) was found in the nucleus, but the distribution within the nucleus was more similar to the protein in cells that do not express DSRed2-AtCPSF30 (Figure 5, panel B). Additionally, GFP-AtCPSF73(I) could also be found in cytoplasmic foci that also contained the DSRed2-m4 variant (Figure 5, panels H and I). In cells co-expressing the DSRed2-m9 mutant and GFP-AtCPSF73(I) proteins, GFP-AtCPSF73(I) was found predominantly in the nucleus, and the DSRed2-m9 protein in the cytoplasm (Figure 5, panels J-L), suggestive of a lack of any interaction between these proteins. These results indicate that AtCPSF30 interacts with AtCPSF73(I) in a manner that promotes a substantial nuclear localization of AtCPSF30, and that the nuclear localization of AtCPSF30 in AtCPSF73(I)-expressing cells requires the N- and C-terminal domains of AtCPSF30.

**Figure 6:**
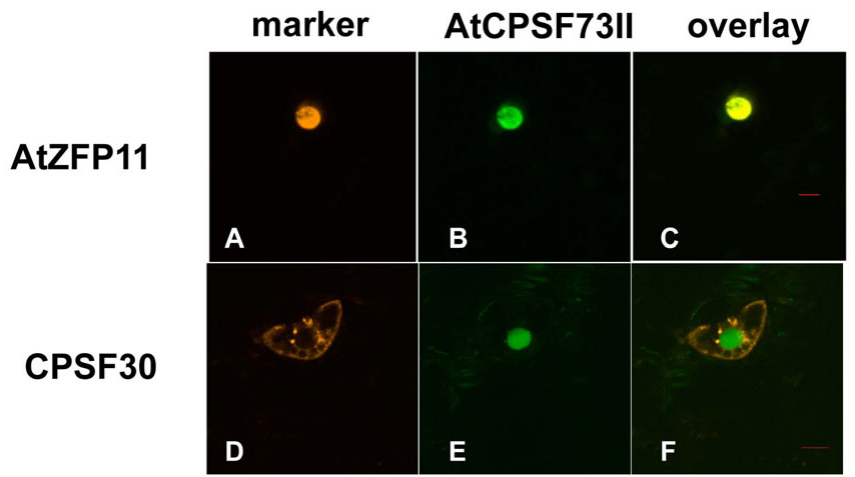
**Localization of AtCPSF30 in cells that also express AtCPSF73(II)**. GFP-AtCPSF73(II) was co-expressed with DSRed2-AtZFP11 (panels A-C) or DSRed2-AtCPSF30 (panels D-F). The column labeled "CPSF73-II" denotes the images of the GFP-AtCPSF73(II) fusion protein, and the column labeled "marker" denotes either the DSRed2-AtZFP11 marker (panel A) or DSRed2-AtCPSF30 (panel D). Red bars in the overlay panels are 10 μm size markers.

Consistent with a previous study [30], GFP-AtCPSF73(II) localized to the nucleus when co-expressed with the nuclear marker (Figure 6, panels A-C). Co-expression of GFP-AtCPSF73(II) with DSRed2-AtCPSF30 did not alter this distribution (Figure 6, panels D-F). Moreover, the cytoplasmic location of DSRed2-AtCPSF30 was not affected by co-expression with GFP-AtCPSF73(II); in particular, no DSRed2-AtCPSF30 could be seen in nuclei. This result is consistent with other results [18] that indicated that these two proteins do not interact.

## Discussion

### On the nature of CPSF in plants

Messenger RNA 3' end formation occurs in the nucleus, and subunits of the complex that mediate this process are expected to reside within the nucleus. The subject of this study, AtCPSF30, can be identified in *Arabidopsis *nuclear extracts and co-purifies with immunoprecipitated AtCPSF100 [14]. Thus, it was surprising that this protein, when over-expressed as DSRed2 or GFP fusions, was not seen in the nucleus. Taken together, these studies indicate that AtCPSF30 itself does not possess nuclear localization information, but rather that it is retained in the nucleus as a consequence of its association with other polyadenylation factor subunits. This hypothesis is supported by the nuclear localization of DSRed2-AtCPSF30 when co-expressed with GFP-AtCPSF160 (Figure 2) or GFP-AtCPSF73(I) (Figure 5). In contrast, co-expression of GFP-AtCPSF100 with DSRed2-AtCPSF30 causes a re-distribution, not of DSRed2-AtCPSF30 (as is the case with GFP-AtCPSF160), but rather of GFP-AtCPSF100 (Figure 4). This result suggests that the AtCPSF100-AtCPSF30 interaction affects the functioning of the nuclear localization information that is inherent in AtCPSF100, perhaps owing to physical contacts between AtCPSF30 and the part of AtCPSF100 that functions as a nuclear localization signal.

Taken together, the patterns of localization seen when AtCPSF30 is co-expressed with other CPSF subunits raise interesting possibilities regarding the organization and functioning of CPSF in plants. The observation that co-expressed AtCPSF30 and AtCPSF100 accumulate largely in the cytoplasm suggests that the nuclear CPSF complex may be organized such that AtCPSF30 is not in contact with AtCPSF100. This in turn raises the possibility that the protein-protein contacts in the nuclear CPSF complex are but a subset of those that have been identified in two-hybrid screens and by direct biochemical assay [13,18,30,31], and lends itself to the idea that CPSF may undergo one or more structural rearrangements in the course of pre-mRNA processing.

Alternatively, the combination of contacts that are implied by the results of this study (between AtCPSF30 and each of AtCPSF160, AtCPSF100, and AtCPSF73(I), respectively) may be simultaneously in force. This would indicate a hierarchy of functionality of nuclear localization information, such that AtCPSF100 would remain in the nucleus even though it associates with a protein (AtCPSF30) that interferes with nuclear localization outside of the context of the complete polyadenylation complex. For example, AtCPSF160 may supersede the effects of AtCPSF30 on the localization of AtCPSF100, either via the interaction between this subunit and the C-terminus of AtCPSF30, or through other interactions such as those between AtCPSF100, AtCPSF73(I) and AtCPSF160 described before [18].

*Arabidopsis *possesses two putative homologs of CPSF73, AtCPSF73(I) and AtCPSF73(II) [18,30,32]. AtCPSF73(I) is more closely related to the canonical CPSF73 as well as the yeast homolog Ysh1 [30], while AtCPSF73(II) is more distantly related [32]. These two proteins may be distinguished by their differing physiological roles; alterations of AtCPSF73(II) expression affect the development of the female gamete in *Arabidopsis *[32], whereas changes in AtCPSF73(I) expression lead to male sterility [30]. The results presented in this study reveal a biochemical difference between the two CPSF73 homologs, in that AtCPSF73(I) interacts with AtCPSF30 so as to promote nuclear localization of the latter protein, while AtCPSF73(II) appears not to interact with AtCPSF30. Whether these differences are the basis for the different physiological functions of the two proteins remains to be determined. However, the possibility attendant with the differences in interactions is supportive of models in which different CPSF complexes exist in plants, complexes that may play specific developmental roles.

These models are not exclusive of one another, and may be combined to yield other variations. The results that inspire them, however, reinforce the suggestion that the nuclear CPSF is a dynamic complex with numerous interesting subtleties.

### A refined functional map of AtCPSF30 – implications for the functioning of the polyadenylation complex

**Figure 7:**
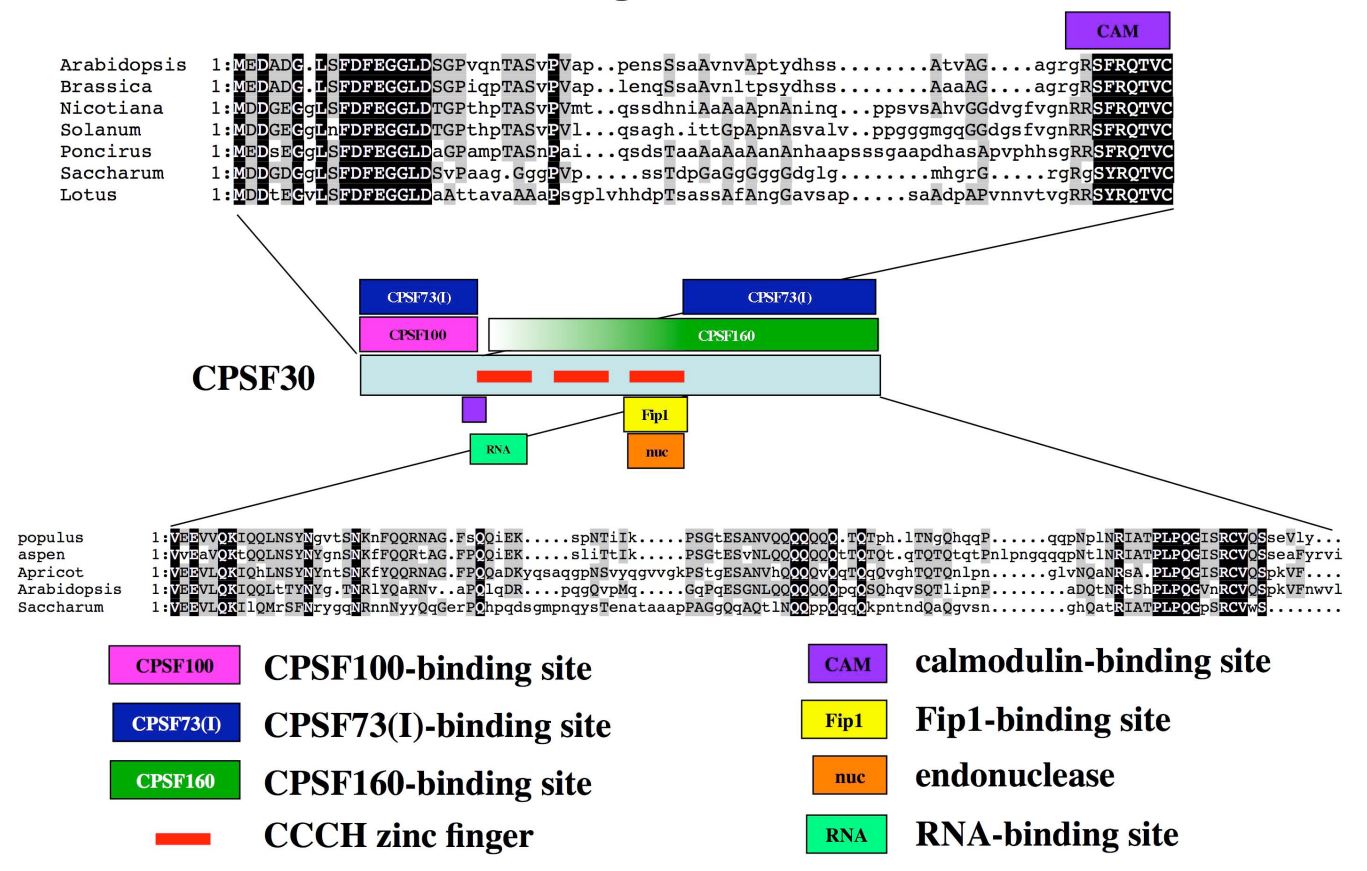
**Functional map of the *Arabidopsis *CPSF30 drawn from other studies **[13,14]**and this report**. A one-dimensional representation of the amino acid sequence (light blue box) is shown in the center of the figure. The color-coding of the various motifs is described at the bottom of the figure. Amino acid sequence alignments of the plant-specific N-terminal and C-terminal domains are shown above and below the functional map of AtCPSF30, respectively. Accession numbers that correspond to the N-terminal sequences: Brassica – CD828769; Nicotiana – CK286112; Solanum – CO502465; Poncirus – CV705634; cane – CA096547; Lotus – BP037936. Accession numbers that correspond to the C-terminal sequences: populus – CV243319; aspen – CK088987; Apricot – CV053103; *Arabidopsis *– AY140901.

AtCPSF30 consists of three identifiable domains (Figure 7), demarcated by the central core of three CCCH-type zinc finger motifs [13,14]. To date, most of the functionality of the protein has been associated with the central zinc finger core. Thus, the first of the three CCCH motifs is responsible for the bulk of the RNA-binding activity of the protein [13,14], and the third CCCH motif is required for both its endonuclease activity and the interactions of the protein with Fip1 [13]. Based on the results of this study, it is apparent that the N-terminal domain is important for the interaction with AtCPSF100 and AtCPSF73(I), and the C-terminus is needed for the interaction with AtCPSF160 and to a lesser extent with AtCPSF73(I). With respect to the AtCPSF30-AtCPSF73(I) interaction, it would seem as if the two contacts implied by the results described here have differing strengths; thus, deletion of the C-terminal domain eliminates the re-distribution of AtCPSF30 into the nucleus but has a less-perceptible effect on the co-localization of the two proteins in the cytoplasm, while deletion of the N-terminus eliminates all discernible interactions between the two proteins.

The interaction between AtCPSF30 and AtCPSF73(I) that is implicit in the results presented here was unanticipated, as it was not observed in a previous two-hybrid mapping of interactions between *Arabidopsis *polyadenylation factor subunits [18]. The reasons for this discrepancy are not clear, but most likely have something to do with the differences in output provided by the two-hybrid assay [18] and co-localization studies. Given that two-hybrid assays are known to yield false negatives [33,34], it is reasonable to assign one more interaction to the hub of interactions that includes AtCPSF30.

AtCPSF30 has a calmodulin-binding site that is located at the junction of the N-terminal domain and the first CCCH zinc finger motif [14]. It is possible that calmodulin may affect the interactions of AtCPSF30 with AtCPSF100 or AtCPSF73(I), and thus provide a link between calcium signaling and the structure of the polyadenylation complex. It is thus of interest to note that AtCPSF30 has been implicated in the responses of *Arabidopsis *to reactive oxygen species [16]; it is tempting to speculate that calmodulin-mediated remodeling of the polyadenylation complex is involved in this function.

The N- and C-terminal domains of AtCPSF30 are absent from their animal and yeast counterparts, and are highly variable in plant CPSF30 orthologs [14]. The involvement of evolutionarily-variable domains in interactions with other CPSF subunits is interesting, because analogous interactions do not seem likely in animals and yeast (since the respective CPSF30 proteins lack these domains). This suggests that the mechanisms by which CPSF30 is held in the CPSF complex may differ in different organisms. This may have implications for the evolution and function of the polyadenylation complex, especially pertaining to regulated and alternative polyadenylation. For example, CPSF30 is the focus of regulatory mechanisms in animals [35-37] and plants [16]; our results raise the possibility that a similar strategy for posttranscriptional regulation – alternative polyadenylation – may be achieved by somewhat different molecular mechanisms in different organisms, and that these different mechanisms could conceivably involve interactions between CPSF30 and other CPSF subunits.

Additionally, the fact that CPSF30 is a core CPSF subunit in eukaryotes indicates that evolutionarily-conserved interactions with proteins other than canonical CPSF subunits may be integral in the association of CPSF30 with the polyadenylation complex. One such interaction is that between CPSF30 and Fip1, another core CPSF subunit [38]. This interaction is conserved in animals, yeast, and plants [13,15,31,38,39]. Moreover, in yeast and plants, Fip1 binds to the same zinc finger motif of CPSF30 [13,15], consistent with an evolutionarily-conserved role for this interaction. While more work is needed to reconcile these various reports, these considerations bring to the fore the CPSF30-Fip1 interaction as one of central importance for the polyadenylation complex.

### A cytoplasmic CPSF in plants?

The cytoplasmic locations of AtCPSF30, AtCPSF100, and AtCPSF73(I) seen in this study are provocative in that they suggest that plants may possess a cytoplasmic complex that includes CPSF subunits. Of course, these cytoplasmic distributions may have limited physiological relevance, as they may be nothing more than reflections of the locations of proteins that are expressed far above levels usually seen in the cell. The association of AtCPSF30 with the ER, the site of protein degradation induced by the unfolded protein response [40-43] is consistent with this possibility. However, given that the cytoplasmic colocalization of AtCPSF100 seems to require an intact AtCPSF30 capable of engaging in protein-protein interactions, it seems unlikely that overexpressed AtCPSF30 or AtCPSF100 are unfolded or otherwise denatured in a manner that might trigger an unfolded protein response. Generally speaking, the promoters used in this study do not lead to high enough expression levels to cause rampant protein mis-localization or degradation [44]. Also, the co-localization of AtCPSF30 and Dcp2 in the cytoplasm is hard to rationalize in this context. However, we cannot completely rule out this possibility.

The alternative is that the cytoplasmic locations of CPSF subunits that we describe are indications of the existence, in plants, of a cytoplasmic CPSF complex. In animals, cytoplasmic forms of CPSF have been identified and characterized. For example, in *Xenopus *oocytes, CPSF functions in the cytoplasm as part of the complex that regulates the polyadenylation and deadenylation of stored cytoplasmic mRNAs [45]. It may be that the cytoplasmic CPSF-containing foci reported here are analogous in some ways to the *Xenopus *cytoplasmic polyadenylation complex.

Yet another alternative is suggested by the observation that AtCPSF30 co-localizes with AtDcp2 (Figure 1). In animals and yeast, Dcp2 is a component of the so-called processing body (or P-body), a dynamic organelle that is involved in mRNA storage, decapping, and degradation. In plants, Dcp2 occurs in a cytoplasmic location along with Dcp1 and VARICOSE, a WD repeat-containing protein [28,29]. These foci are likely the *Arabidopsis *counterparts of P-bodies, locations of mRNA decapping and breakdown. The association of AtCPSF30 with Dcp2 is interesting, as it insinuates CPSF into the mRNA-degrading system. Such a possibility lends itself to numerous future studies, and may lead to a more integrated view of RNA metabolism in plants.

## Conclusion

In conclusion, the results presented in this paper reveal that the distribution of the *Arabidopsis *CPSF30 within the cell is defined by its interactions with other CPSF subunits, and that this protein in turn affects the distribution of some CPSF subunits. They also show that the interactions between AtCPSF30 and other CPSF subunits involve plant-specific domains of AtCPSF30, indicative of a degree of evolutionary novelty in the functioning of AtCPSF30. These results lend themselves to interesting possibilities for the structure and functioning of CPSF, and they raise new possibilities for the roles of CPSF in RNA processing and metabolism in plants.

## Methods

### Materials

MS salts, restriction enzymes and T4-DNA ligase were obtained from American Allied Biochemical, Inc. (Aurora, CO, USA) or Invitrogen (Carlsbad, California). Plant gene expression vectors carrying genes encoding the GFP-AtCPSF160, GFP-AtCPSF100, GFP-AtCPSF73(I), and GFP-AtCPSF73(II) proteins were obtained from Dr. Q. Q. Li (Miami University, Oxford, OH). The AtCPSF160 and AtCPSF100 plasmids are derivatives of pMDC43 [46] while the AtCPSF73(I) and AtCPSF73(II) plasmids are pKYLX80 derivatives [22,30]. mGFP5-ER, the pSITE derivative encoding the endoplasmic reticulum marker [47], was obtained from Dr. M. M. Goodin (Dept. of Plant Pathology, University of Kentucky). The plasmids encoding the AtZFP11 fusion proteins, used here as a nuclear marker, have been described elsewhere [22]. The mitochondrial marker, consisting of the amino-terminal 60 amino acids of the *Arabidopsis *NDA2 gene product (At2g29990) fused to GFP in a pKYLX80 backbone, has been described [20].

### Recombinant DNA manipulations

Genes encoding fusion proteins containing AtCPSF30, the m4 and m9 mutant derivatives of AtCPSF30 [13,14], and AtDcp2 were assembled using PCR and the appropriate DNA templates and primers; these are listed in Table 2. The same general strategy was used for all of these. PCR products were first cloned into pGEM T-Easy (Promega, Madison, WI, USA) and recombinants identified by restriction enzyme analysis; a representative clone for each construct was confirmed by DNA sequencing and used for further manipulations. The respective protein-coding region was excised with BglII + ApaI; these sites were embedded in the PCR primers in such a way as to permit in-frame fusion of these protein coding regions to the GFP or DSR coding regions in the pGD series of plasmid [19]. The BglII-ApaI fragments were subcloned into suitably-digested pGD vector and recombinants identified by colony PCR; clones for subsequent use were confirmed by DNA sequencing.

**Table 2:** Primers and plasmids used for this study

	**primers**		
Designation	Sequence (5' -> 3') or description	use	Reference(s)

AtCPSF30 5'	AGATCTATGGAGGATGCTGATGGACTT	Cloning of the AtCPSF30 protein-coding region	This study

AtCPSF30 3'	CCGGAGATCTATGTCGGGCCTCCATCGATC	Cloning of the AtCPSF30 protein-coding region	This study

C-ter 30 5' (m9)	AGATCTGGAGCTGGGAGGGGTAGAAGTTTCCGTCAA	Cloning of the AtCPSF30 C-terminal protein-coding region	This study

N-ter 30 3' (m4)	GGGCCCAGGTCCAGGAAGCTTTGCATGCCTGTACCGACA	Cloning of the AtCPSF30 N-terminal protein-coding region	This study

AtDcp2 5'	CCGGAGATCTATGTCGGGCCTCCATCGATC3	Cloning of the AtDcp2 protein-coding region	This study

AtDcp2 3'	AATTGGGCCCCAAACTGACCAGTCAAGCTGAATTACCAG	Cloning of the AtDcp2 protein-coding region	This study

	**plasmids**		

designation	source	use	Reference(s)

Salk clone U61209	ABRC; corresponds to At5g13570	Template for amplification of AtDcp2 sequences	

pMAL-AtCPSF30	Hunt laboratory	Template for amplification of AtCPSF30 sequences	[14]

pMDC43C1-GFP::AtCPSF160, pMDC43C1-GFP::AtCPSF100, pMDC43C1-GFP-AtCPSF73(I) and GFP-AtCPSF73(II)	Dr. Q. Q. Li (Miami University, Oxford, OH)	Plasmids for the expression of GFP fused *Arabidopsis *CPSF factors 160, 100, 73CII and 73CI	[30]

pGD RFP, pGD RFP-NLS,	Dr. Michael Goodin (University of Kentucky, Lexington, KY)	pGD RFP vector was used to express different CPSF 30 clones; pGD RFP-NLS was used as control in GFP fusion studies.	[19]

pKLX80- AtZFP11-NLS::GFP, pKLX80-CoxII::GFP, pKLX80-ER::GFP	Dinkins laboratory		[20,22]

### Microprojectile bombardment

*Nicotiana tabacum *L. cv. KY160 (University of Kentucky Tobacco Breeding Program) was grown in the greenhouse. The abaxial surface of leaves from four week-old plants were bombarded with mixtures of plasmids as indicated in the text using a PDS1000 DuPont BioRad Micro projectile delivery system (BioRad Laboratories, Hercules, CA). Just before bombardment leaves were cut at the petiole and placed in a Petri dish on a moist filter paper. Gene gun bombardment was done as described [48]. Briefly, for nine shots, 12.5 μg of DNA was used to coat 7.5 mg of 1.0 μm gold spheres; this translates to 0.83 mg of gold particles and 1.38 ug of DNA per bombardment. Leaves were bombarded at 1,100 psi, helium gas pressure under a 27 in of Hg vacuum, at a shooting distance of 11 cm from rupture disk to target tissue. Immediately after bombardment, tobacco leaves were placed on T- medium for 48 hrs. All plasmid DNAs used in the bombardment studies were prepared using Wizard^R ^Plus SV Minipreps Purification system (Promega, Madison, WI, USA).

In one experiment, nuclear DNA was identified by staining with Hoechst 33258 (Invitrogen/Molecular Probes, Eugene, OR). Hoechst stain was dissolved in 10 mM Tris-1 mM EDTA buffer to a concentration of 0.01 mg/ml. Leaf pieces were immersed in the stain for 10 minutes in the presence of Tween-20 0(.01%). After staining the leaf pieces were washed in Tris-EDTA buffer 3 times and nuclei were visualized and photographed as described [49].

### Confocal laser scanning microscopy

Confocal laser scanning micrographs were obtained using an Olympus Fluorview™ FV1000 microscope (Olympus America, Inc, Center Valley, PA.). GFP was excited at 488 nm with an argon laser, and emission light was captured through a band-pass emission filter (BP485-515). DSRed2 was excited at 543 nm with a helium-neon laser, and emission light was captured through a band-pass emission filter (BP520-590). Chlorophyll autofluorescence was visualized using the GFP laser (488 nm) for excitation and monitoring light above 650 nm using a 650 nm bypass filter. All images were visualized using at 40× objective lens and images were captured using the Olympus Fluorview™ software (ver 1.4a) and each channel was sequentially scanned by line. Single focal planes with representative distributions were chosen for presentation. The numbers of cells assessed for each combination of constructs is given in Additional File 2.

### Sequence analysis

Amino acid sequence data were analyzed using Vector NTI software (Informax). Multiple amino acid sequence alignments were performed using Clustal X 1.83 and displayed using MacBoxshade.

## Abbreviations

ABRC: Arabidopsis Biological Resource Center; CPSF: Cleavage and Polyadenylation Specificity Factor; CstF: Cleavage Stimulatory Factor; DsRed2: red fluorescent protein 2-; GFP: green fluorescent protein.

## Authors' contributions

SR performed the recombinant DNA manipulations and the transfection studies. RD assisted with the transfection studies and directed the microscopy work. AGH conceived of the study, oversaw the experimental design, and wrote the manuscript. All authors read and approved the final manuscript.

## Supplementary Material

Additional file 1 **Location and movement of AtCPSF30**. This file shows a cell bombarded with DSR-AtCPSF30 and GFP-AtZFP11 (the nuclear marker), with both markers and chloroplasts visualized and displayed. Images were captured every 2.213 seconds, with a total duration of 58 sec (26 images). Click here for file

Additional file 2 **Summary of confocal microscopy experiments**. This file is a tabular summation of the confocal microscopy experiments. Click here for file

## References

[B1] Proudfoot N, O'Sullivan J (2002). Polyadenylation: a tail of two complexes. Curr Biol.

[B2] Wahle E, Ruegsegger U (1999). 3'-End processing of pre-mRNA in eukaryotes. FEMS Microbiol Rev.

[B3] Zhao J, Hyman L, Moore C (1999). Formation of mRNA 3' ends in eukaryotes: mechanism, regulation, and interrelationships with other steps in mRNA synthesis. Microbiol Mol Biol Rev.

[B4] Murthy KG, Manley JL (1995). The 160-kD subunit of human cleavage-polyadenylation specificity factor coordinates pre-mRNA 3'-end formation. Genes Dev.

[B5] Beyer K, Dandekar T, Keller W (1997). RNA ligands selected by cleavage stimulation factor contain distinct sequence motifs that function as downstream elements in 3'-end processing of pre-mRNA. J Biol Chem.

[B6] MacDonald CC, Wilusz J, Shenk T (1994). The 64-kilodalton subunit of the CstF polyadenylation factor binds to pre-mRNAs downstream of the cleavage site and influences cleavage site location. Mol Cell Biol.

[B7] Takagaki Y, Manley JL (1997). RNA recognition by the human polyadenylation factor CstF. Mol Cell Biol.

[B8] Kuhn U, Wahle E (2004). Structure and function of poly(A) binding proteins. Biochim Biophys Acta.

[B9] Barabino SM, Ohnacker M, Keller W (2000). Distinct roles of two Yth1p domains in 3'-end cleavage and polyadenylation of yeast pre-mRNAs. Embo J.

[B10] Barabino SM, Hubner W, Jenny A, Minvielle-Sebastia L, Keller W (1997). The 30-kD subunit of mammalian cleavage and polyadenylation specificity factor and its yeast homolog are RNA-binding zinc finger proteins. Genes Dev.

[B11] Bai C, Tolias PP (1998). Drosophila clipper/CPSF 30K is a post-transcriptionally regulated nuclear protein that binds RNA containing GC clusters. Nucleic Acids Res.

[B12] Bai C, Tolias PP (1996). Cleavage of RNA hairpins mediated by a developmentally regulated CCCH zinc finger protein. Mol Cell Biol.

[B13] Addepalli B, Hunt AG (2007). A novel endonuclease activity associated with the Arabidopsis ortholog of the 30-kDa subunit of cleavage and polyadenylation specificity factor. Nucleic Acids Res.

[B14] Delaney KJ, Xu R, Zhang J, Li QQ, Yun KY, Falcone DL, Hunt AG (2006). Calmodulin interacts with and regulates the RNA-binding activity of an Arabidopsis polyadenylation factor subunit. Plant Physiol.

[B15] Tacahashi Y, Helmling S, Moore CL (2003). Functional dissection of the zinc finger and flanking domains of the Yth1 cleavage/polyadenylation factor. Nucleic Acids Res.

[B16] Zhang J, Addepalli B, Yun K-Y, Hunt AG, Xu R, Rao S, Li QQ, Falcone DL (2008). A Polyadenylation Factor Subunit Implicated in Regulating Oxidative Signaling in Arabidopsis thaliana. PLoS ONE.

[B17] Addepalli B, Hunt AG (2008). Redox and heavy metal effects on the biochemical activities of an Arabidopsis polyadenylation factor subunit. Arch Biochem Biophys.

[B18] Hunt AG, Xu R, Addepalli B, Rao S, Forbes KP, Meeks LR, Xing D, Mo M, Zhao H, Bandyopadhyay A (2008). Arabidopsis mRNA polyadenylation machinery: comprehensive analysis of protein-protein interactions and gene expression profiling. BMC Genomics.

[B19] Goodin MM, Dietzgen RG, Schichnes D, Ruzin S, Jackson AO (2002). pGD vectors: versatile tools for the expression of green and red fluorescent protein fusions in agroinfiltrated plant leaves. Plant J.

[B20] Dinkins RD, Majee SM, Nayak NR, Martin D, Xu Q, Belcastro MP, Houtz RL, Beach CM, Downie AB (2008). Changing transcriptional initiation sites and alternative 5'- and 3'-splice site selection of the first intron deploys Arabidopsis PROTEIN ISOASPARTYL METHYLTRANSFERASE2 variants to different subcellular compartments. Plant J.

[B21] Haseloff J, Siemering KR, Prasher DC, Hodge S (1997). Removal of a cryptic intron and subcellular localization of green fluorescent protein are required to mark transgenic Arabidopsis plants brightly. Proc Natl Acad Sci USA.

[B22] Dinkins RD, Pflipsen C, Collins GB (2003). Expression and deletion analysis of an Arabidopsis SUPERMAN-like zinc finger gene. Plant Sci.

[B23] Deng M, Bragg JN, Ruzin S, Schichnes D, King D, Goodin MM, Jackson AO (2007). Role of the sonchus yellow net virus N protein in formation of nuclear viroplasms. J Virol.

[B24] Goodin MM, Austin J, Tobias R, Fujita M, Morales C, Jackson AO (2001). Interactions and nuclear import of the N and P proteins of sonchus yellow net virus, a plant nucleorhabdovirus. J Virol.

[B25] Parker R, Sheth U (2007). P bodies and the control of mRNA translation and degradation. Mol Cell.

[B26] Eulalio A, Behm-Ansmant I, Izaurralde E (2007). P bodies: at the crossroads of post-transcriptional pathways. Nat Rev Mol Cell Biol.

[B27] Gunawardana D, Cheng HC, Gayler KR (2008). Identification of functional domains in Arabidopsis thaliana mRNA decapping enzyme (AtDcp2). Nucleic Acids Res.

[B28] Iwasaki S, Takeda A, Motose H, Watanabe Y (2007). Characterization of Arabidopsis decapping proteins AtDCP1 and AtDCP2, which are essential for post-embryonic development. FEBS Lett.

[B29] Xu J, Yang JY, Niu QW, Chua NH (2006). Arabidopsis DCP2, DCP1, and VARICOSE form a decapping complex required for postembryonic development. Plant Cell.

[B30] Xu R, Zhao H, Dinkins RD, Cheng X, Carberry G, Li QQ (2006). The 73 kD subunit of the cleavage and polyadenylation specificity factor (CPSF) complex affects reproductive development in Arabidopsis. Plant Mol Biol.

[B31] Forbes KP, Addepalli B, Hunt AG (2006). An Arabidopsis Fip1 homolog interacts with RNA and provides conceptual links with a number of other polyadenylation factor subunits. J Biol Chem.

[B32] Xu R, Ye X, Quinn Li Q (2004). AtCPSF73-II gene encoding an Arabidopsis homolog of CPSF 73 kDa subunit is critical for early embryo development. Gene.

[B33] Huang H, Bader JS (2009). Precision and recall estimates for two-hybrid screens. Bioinformatics.

[B34] Huang H, Jedynak BM, Bader JS (2007). Where have all the interactions gone? Estimating the coverage of two-hybrid protein interaction maps. PLoS Comput Biol.

[B35] Nemeroff ME, Barabino SM, Li Y, Keller W, Krug RM (1998). Influenza virus NS1 protein interacts with the cellular 30 kDa subunit of CPSF and inhibits 3'end formation of cellular pre-mRNAs. Mol Cell.

[B36] Twu KY, Kuo RL, Marklund J, Krug RM (2007). The H5N1 influenza virus NS genes selected after 1998 enhance virus replication in mammalian cells. J Virol.

[B37] Twu KY, Noah DL, Rao P, Kuo RL, Krug RM (2006). The CPSF30 binding site on the NS1A protein of influenza A virus is a potential antiviral target. J Virol.

[B38] Kaufmann I, Martin G, Friedlein A, Langen H, Keller W (2004). Human Fip1 is a subunit of CPSF that binds to U-rich RNA elements and stimulates poly(A) polymerase. EMBO J.

[B39] Helmling S, Zhelkovsky A, Moore CL (2001). Fip1 regulates the activity of Poly(A) polymerase through multiple interactions. Mol Cell Biol.

[B40] Malhotra JD, Kaufman RJ (2007). The endoplasmic reticulum and the unfolded protein response. Semin Cell Dev Biol.

[B41] Schroder M (2008). Endoplasmic reticulum stress responses. Cell Mol Life Sci.

[B42] Sitia R, Molteni SN (2004). Stress, protein (mis)folding, and signaling: the redox connection. Sci STKE.

[B43] Ron D, Walter P (2007). Signal integration in the endoplasmic reticulum unfolded protein response. Nat Rev Mol Cell Biol.

[B44] Goodin MM, Chakrabarty R, Banerjee R, Yelton S, Debolt S (2007). New gateways to discovery. Plant Physiol.

[B45] Dickson KS, Bilger A, Ballantyne S, Wickens MP (1999). The cleavage and polyadenylation specificity factor in Xenopus laevis oocytes is a cytoplasmic factor involved in regulated polyadenylation. Mol Cell Biol.

[B46] Curtis MD, Grossniklaus U (2003). A Gateway Cloning Vector Set for High-Throughput Functional Analysis of Genes in Planta. Plant Physiol.

[B47] Chakrabarty R, Banerjee R, Chung SM, Farman M, Citovsky V, Hogenhout SA, Tzfira T, Goodin M (2007). PSITE vectors for stable integration or transient expression of autofluorescent protein fusions in plants: probing Nicotiana benthamiana-virus interactions. Mol Plant Microbe Interact.

[B48] Dinkins RD, Reddy MSS, Meurer CA, Redmond CT, Collins GB, Jaiwal PK, Singh RP (2004). Recent advances in soybean transformation. Applied Genetics of Leguminosae Biotechnology.

[B49] Meadows MG, Potrykus I (1981). Hoechst 33258 as a vital stain for plant cell protoplasts. Plant Cell Reports.

